# MiR-215, an activator of the CTNNBIP1/β-catenin pathway, is a marker of poor prognosis in human glioma

**DOI:** 10.18632/oncotarget.4622

**Published:** 2015-07-22

**Authors:** Yong-Qing Tong, Bei Liu, Hong-Yun Zheng, Jian Gu, Hang Liu, Feng Li, Bi-Hua Tan, Melanie Hartman, Chunhua Song, Yan Li

**Affiliations:** ^1^ Department of Clinical Laboratory, Renmin Hospital of Wuhan University, Wuhan 430060, PR China; ^2^ Clinical Molecular Diagnostic Center, Renmin Hospital of Wuhan University, Wuhan 430060, PR China; ^3^ Department of Pathology Affiliated Tianyou Hospital of Wuhan University of Science and Technology, Wuhan 430064, PR China; ^4^ Pennsylvania State University College of Medicine and Hershey Medical Center, Hershey, Pennsylvania 17033, USA

**Keywords:** miR-215, glioma, prognosis, CTNNBIP1, TGF-β1

## Abstract

MicroRNA-215 (miR-215) promotes tumor growth in various human malignancies. However, its role has not yet been determined in human glioma. Here, we found that levels of miR-215 were higher in glioma tissues than in corresponding non-neoplastic brain tissue. High miR-215 expression was correlated with higher World Health Organization (WHO) grades and shorter overall survival. Multivariate and univariate analysis indicated that miR-215 expression was an independent prognostic factor. We also found that TGF-beta1, phosphorylated beta-catenin, alpha-SMA, and fibronectin were increased in glioma tissues. Additionally, CTNNBIP1, a direct target of miR-215, was decreased in glioma compared to adjacent normal tissue. These data indicate that miR-215 activates Wnt/β-catenin signaling by increasing β-catenin phosphorylation, α-SMA expression, and fibronectin expression. It promotes TGF-β1-induced oncogenesis by suppressing CTNNBIP1 in glioma. In summary, miR-215 is overexpressed in human glioma, is involved in TGF-β1-induced oncogenesis, and can be used as a marker of poor prognosis in glioma patients.

## INTRODUCTION

Gliomas account for over 50% of brain tumors and are the most serious form of central nervous system tumors that affect adults [1]. The most common grading system for gliomas is the World Health Organization (WHO) classification of 2007, under which tumors are graded from I (least advanced disease / best prognosis) to IV (most advanced disease / worst prognosis) [2].

Gliomas originate from astrocytes, oligodendrocytes, or ependymal cells with tumors usually displaying a mixture of two or three of these different glial cell types. Tumor heterogeneity results in aggressive tumors that are resistant to treatment. Glioblastoma multiforme (GBM) is the most lethal primary malignancy of the central nervous system (CNS) because it is extremely aggressive [3]. Most gliomas are highly malignant with a poor prognosis [4]. It is reported that the overall median survival in patients with glioma is about 14.6 months with a two-year survival rate at 30% [5]. So far, there has been little progress in improving treatment or patient survival. Therefore, it is important and valuable to understand the molecular biology of gliomas to develop novel therapeutics for this disease.

MicroRNAs (miRNAs) are single stranded, evolutionarily conserved, RNA molecules and are around 22 nucleotides in length. They function post-transcriptionally by binding with partial complementarity to the mRNA of target genes. miRNAs regulate the expression of genes that play key roles in both healthy and diseased states [6]. On average, one miRNA is predicted to negatively regulate the expression of hundreds of target genes. It is also estimated that more than one-third of human genes are regulated by miRNAs [7] with more than 1900 miRNA genes identified in the human genome. Many miRNAs have been implicated in the pathogenesis, diagnosis, and therapy of several cancers, including pancreatic cancer, cervical cancer, and ovarian cancer. Many miRNAs have been identified in gliomas, including miR-145 [8], miR-137 [9], and miR-134 [10]. These miRNAs are involved in cell proliferation, cell cycle regulation, apoptosis, tumor invasion, glioma stem cell behavior, and angiogenesis. However, no miRNAs are reported to correlate with the prognosis of human glioma.

MicroRNA-215 (miR-215), a transcript of chromosome 11q13.1, was initially thought to be a positive regulator of p53. It was later found to have oncogenic effects in various tumors. miR-215 is upregulated in gastric cancer and may be a potential biomarker for gastric cancer prognosis [11]. Downregulation of miR-215 expression suppresses gastric cancer cell invasion, suggesting the possibility that expression of miR-215 influences gastric cancer progression. The expression and function of miR-215 in human glioma tissues has yet to be explored. Here we examine miR-215 expression in human glioma and investigate the clinical significance of miR-215 overexpression.

## RESULTS

### miR-215 expression is increased in human glioma tissue

**Figure 1:**
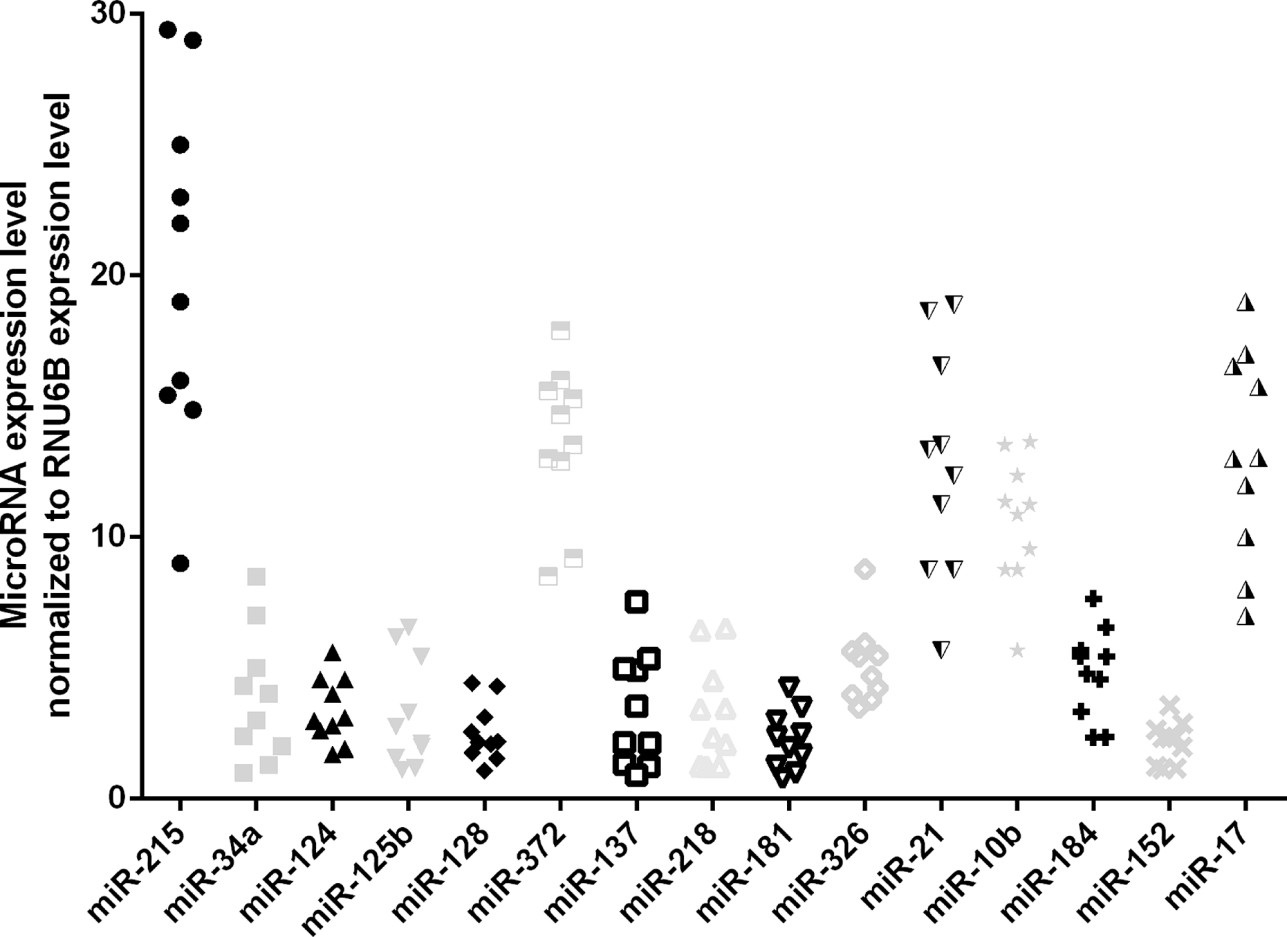
miRNAs expression in glioma tissues The expression level of 15 miRNAs were examined by qRT-PCR assay.

To explore the effect of miRNAs on human gliomas, we measured the expression of miRNAs in human glioma tissue and found that miR-215 is the most highly expressed. miR-372, miR-21, mIR10b, and miR-17 were intermediately expressed, and 10 other miRNAs were expressed at a lower level (Fig. 1). These data indicate an important oncogenic role for miR-215 in human glioma.

**Figure 2:**
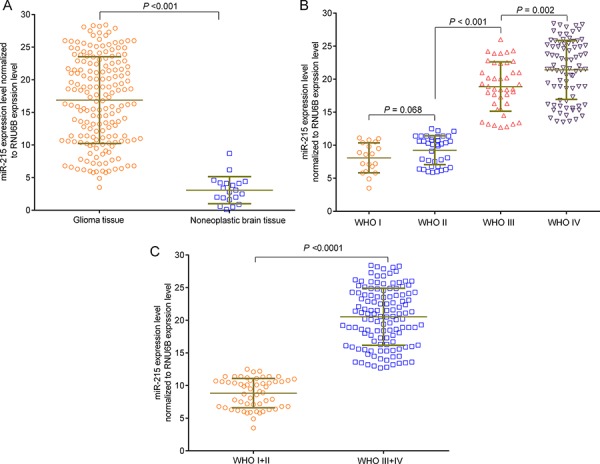
miR-215 overexpression in glioma tissues as detected by qRT-PCR assay **A.** The expression level of miR-215 was higher in tumor tissues than in non-tumor tissues. **B.** The expression levels of miR-215 in different stages of glioma. **C.** The expression level of miR-215 in WHO I+II and WHO III+IV stages of the glioma. The stages were graded with WHO classification criteria.

We further examined miR-215 expression in 179 primary glioma and 20 non-neoplastic brain tissues by quantitative real-time PCR (qRT-PCR). Levels of miR-215 in tumor tissues (16.88 ± 0.50) were higher than that in non-tumor tissues (3.06 ± 0.46) (*P* < 0.001) (Fig. 2A). The gliomas were classified into 4 stages based on WHO criteria, and miR-215 expression was compared between the 4 stages of gliomas. We found increased levels of miR-215 in tumors with advanced staging (WHO I: 8.08 ± 0.52, WHO II: 9.24 ± 0.36, WHO III: 18.87 ± 0.58, WHO IV: 21.41 ± 0.50) (*P* < 0.001) (Fig. 2B). No significant difference in miR-215 expression was found between low-grade gliomas (WHO I and WHO II) (*P* = 0.068) (Fig. 2B). Expression of miR-215 was lower in low-grade gliomas (WHO I + II: 8.850 ± 0.30) compared to high-grade gliomas (WHO III + IV: 20.54 ± 0.39) (*P* < 0.001) (Fig. 2C).

### miR-215 overexpression is associated with clinicopathological characteristics of human gliomas

For statistical analysis, miR-215 expression was divided into high and low expression groups (G4 versus G1–3). We examined the association of miR-215 expression with clinicopathological characteristics in patients with gliomas. We observed a correlation between miR-215 expression and the histopathological grade (Table 1). High expression of miR-215 occurred more frequently in high-grade tumors (III–IV, 65.85%) than in low-grade tumors (I–II, 39.28%; *P* = 0.001). Also, a correlation was observed between high miR-215 expression, tumor dimension, and tumor recurrence or metastasis (*P* = 0.012 and 0.042, respectively). No significant association of miR-215 expression was found with age, gender, or KPS score of the patients in the cohort (all *P* ≥ 0.05, Table 1).

**Table 1:** Association between miR-215 expression and different clinicopathological characteristics of human gliomas

Clinicopathological characteristics	No. of cases	miR-215 expression		*P*
		High [*n* (%)]	Low [*n* (%)]	
**Age**				0.776
**<40**	87	51 (58.62)	36 (41.38)	
**≥40**	92	52 (56.52)	40 (43.48)	
**Gender**				0.719
**Male**	97	57 (58.76)	40 (41.24)	
**Female**	82	46 (56.10)	36 (43.90)	
**WHO Grade**				0.001
**I**	19	7 (36.84)	12 (63.16)	
**II**	37	15 (40.54)	22 (59.46)	
**III**	42	29 (69.05)	13 (30.95)	
**IV**	81	52 (64.20)	29 (35.80)	
**KPS score**^#^				0.403
**<60**	103	62 (60.19)	41 (39.81)	
**≥60**	76	41 (53.95)	35 (46.05)	
**Tumor dimension**^#^				0.012
**<6 cm**	118	60 (50.85)	58 (49.15)	
**≥6 cm**	61	43 (70.49)	18 (29.51)	
**Recurrence or metastasis**				0.042
**Negative**	43	19 (44.19)	24 (55.81)	
**Positive**	136	84 (61.76)	52 (38.24)	

### High miR-215 expression is associated with poor prognosis in patients with gliomas

**Figure 3:**
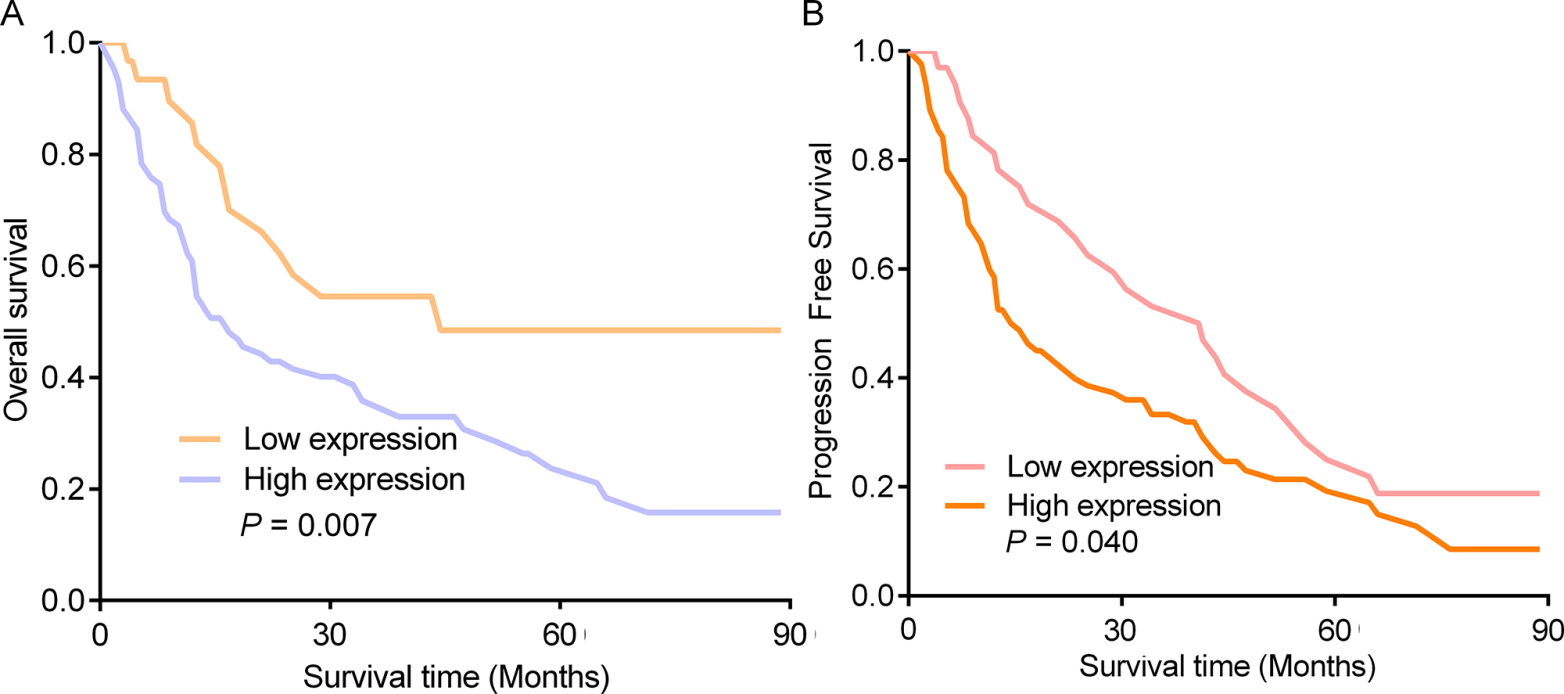
Comparison of Kaplan-Meier survival curves for glioma patients in high and low miR-215 expression groups **A.** The comparison of 5-year overall survival rate of glioma patients with high miR-215 expression with that of patients with low miR-215 expression. **B.** Comparison of 5-year progression free survival rate of glioma patients with high miR-215 expression with that of patients with low miR-215 expression.

In order to understand the prognostic significance of high miR-215 levels, the survival of patients with high miR-215 expression was compared to that of patients with low expression. We found that glioma patients with high miR-215 expression had a shorter median overall survival (OS) than those with low expression (44 months vs. 15 months, *P* = 0.040, Fig. 3A). Also, the median progression free survival was shorter in patients with high expression than those with low expression (41 months vs. 13 months, *P* = 0.007, Fig. 3B). No significant associations were found between the survival rate and gender, age, KPS score, chemotherapy, or radiotherapy.

**Figure 4:**
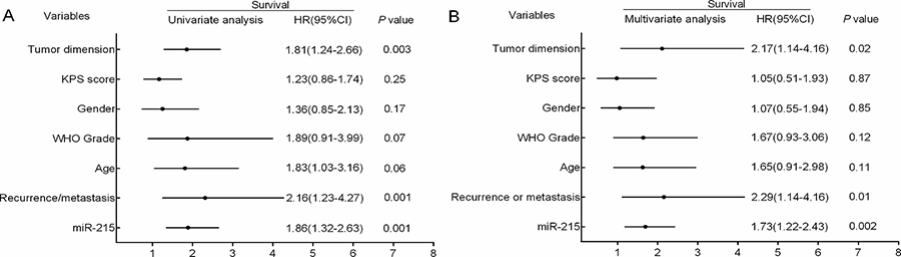
Univariate and multivariate analyses for overall survival rate by Cox regression model **A.** Univariate analysis of the hazard ratios for miR-215 overexpression, tumor dimension, and recurrence or metastasis as independent prognostic factors to predict the overall survival. **B.** Multivariate analysis of the hazard ratios (HRs) for miR-215 overexpression, tumor dimension, and recurrence or metastasis as independent prognostic factors to predict the overall survival. HR, hazard ratio; CI, confidence interval.

Moreover, we analyzed the difference of OS and PFS in different WHO grades. The OS and PFS in grade III and IV are significantly shorter than Grade I (Table 2) or Grade II (data not shown). Furthermore, univariate and multivariate analyses for OS were performed to evaluate whether miR-215 expression levels and clinicopathological features were independent prognostic parameters of patient outcomes. Univariate analysis using the Cox proportional hazards model for all variables showed that high miR-215 expression (*P* = 0.001, 95%CI HR 1.32–2.63), recurrence or metastasis (*P* = 0.001, 95%CI HR 1.23–4.27), and tumor dimension (*P* = 0.003, 95%CI HR 1.24–2.66) were all independent poor prognostic parameters for glioma patients (Fig. 4A).

**Table 2:** Correlation of pathological grade with prognosis in glioma patients

WHO grade	OS (mean ± SD, unit/months)	*P* (vs grade I)	PFS (mean ± SD, unit/months)	*P* (vs grade I)
I	39.27 ± 17.31		31.43 ± 16.03	
II	33.54±11.87	0.1505	28.56 ± 14.94	0.5095
III	25.82±13.59	0.0017	19.98 ± 8.27	0.0005
IV	16.33±9.29	<0.0001	14.92 ± 9.38	<0.0001

Multivariate analysis using the same variables as in the univariate analysis in the cohort confirmed that miR-215 expression (*P* = 0.002, 95%CI HR 1.22–2.43), recurrence or metastasis (*P* = 0.01, 95%CI HR 2.29–4.16), and tumor dimension (*P* = 0.02, 95%CI HR 1.14–4.16) were independent poor prognostic parameters for glioma patients (Fig. 4B). However, no significant association of OS was found with other clinicopathological features including sex, age, and KPS score.

### miR-215 activates β-catenin pathways by decreasing TNNBIP1 expression in gliomas

**Figure 5:**
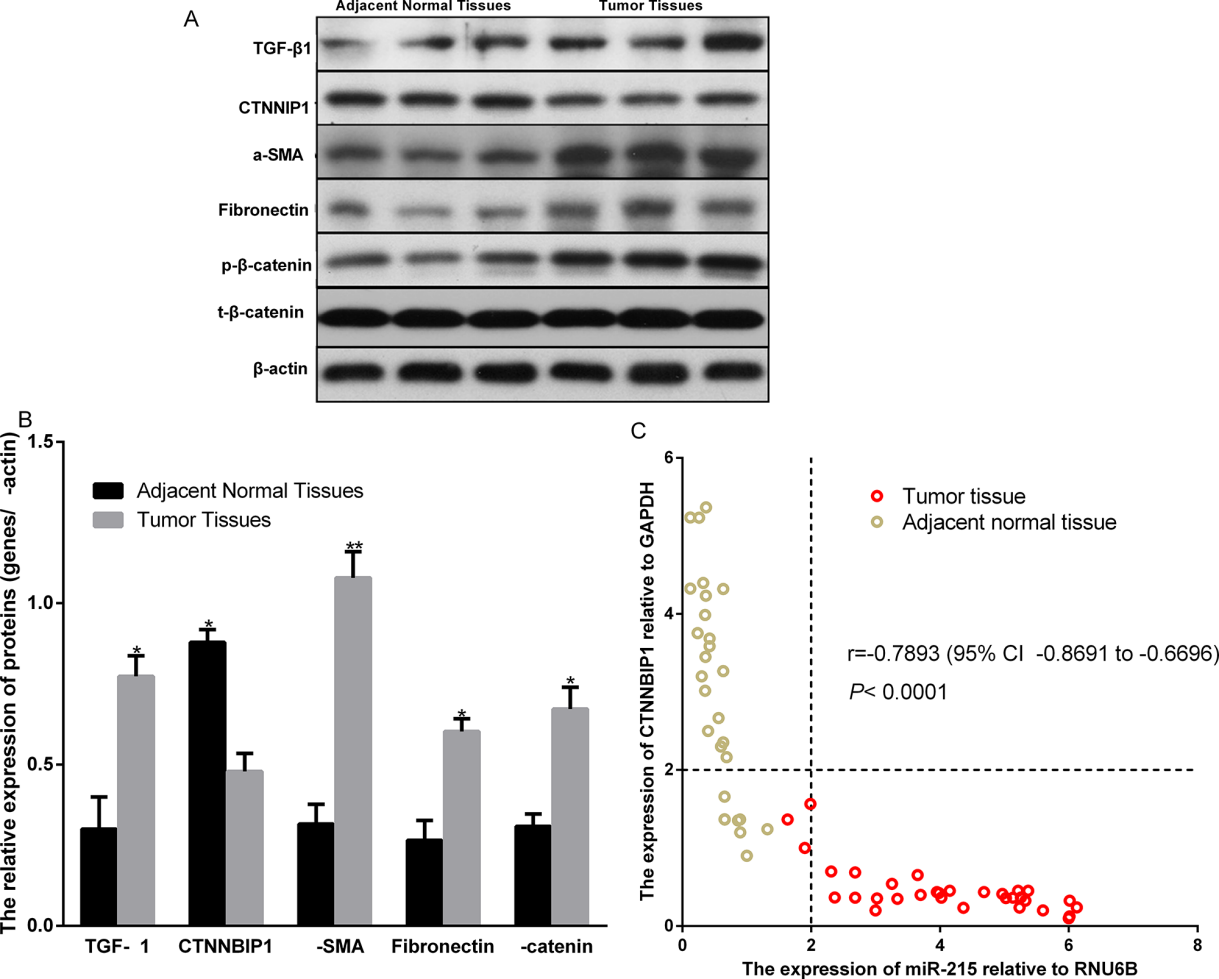
Expression comparison of TGF-β1, CTNNBIP1, α-SMA, and Fibronectin in human glioma tumor tissue and adjacent normal tissue **A–B.** Expression of the indicated proteins in adjacent normal tissue and tumor tissues are showed in A by western blot and in B as quantitated data. **C.** CTNNBIP1 mRNA and miR-215 expression levels in the tumor tissue and adjacent normal tissue are examined using qPCR and their correlation was analyzed by the Pearson correlation method.

Next, we explored the mechanism underlying the oncogenic effect of miR-215. It is reported that CTNNBIP1 is a direct target of miR-215 [12]. miR-215 is a positive regulator of Wnt/β-catenin signaling and prompts a TGF-β1-induced increase of α-SMA and fibronectin specifically by suppression of CTNNBIP1 [12]. TGF-β1 signaling is reported to be dramatically increased in human glioma [13] and α-SMA and fibronectin play important roles in promoting glioma metastasis. We also examined the expression of TGF-β1, β-catenin phosphorylation, CTNNBIP1, α-SMA, and fibronectin in tumor tissue and adjacent normal tissue by Western blot (Fig. 5A). We found that TGF-β1 expression and β-catenin phosphorylation were indeed increased in the glioma tissue compared to adjacent normal tissue (Fig. 5B). We also observed that a-SMA and fibronectin were increased in the tumor tissue compared to adjacent normal tissue (Fig. 5B). Importantly, we observed that the CTNNBIP1 is decreased in the tumor tissue compared to adjacent normal tissue (Fig. 5B). We also examined the mRNA level of CTNNBIP1 in tumor tissue and adjacent normal tissue and compared it with that of miR-215 (Fig. 5C). We found that high CTNNBIP expression was detected in 97% (29/30) of adjacent normal tissue and low CTNNBIP expression was detected in 90% (27/30) of glioma tissues. High miR-215 expression was detected in 90% (27/30) of glioma tissues and low miR-215 expression was detected in 100% (30/30) of adjacent normal tissue (Fig. 5C). These data indicate that CTNNBIP1 has high expression in adjacent normal tissue and low expression in tumor tissue; conversely miR-215 has high expression in tumor tissue and low in adjacent normal tissue. We also analyzed the correlation of CTNNBIP1 expression with that of miR-215. Importantly, CTNNBIP1 expression is negatively correlated with miR-215 expression (*r* = −0.7893 95% CI −0.8691 to −0.6696; *P* < 0.0001) (Fig. 5C).

## DISCUSSION

miRNAs are important players in tumor development and metastasis. We found differentially expressed levels of miRNAs in cancer that targeted key molecules involved in tumor progression [14, 15]. There have been many reports about the expression and function of miRNAs in gliomas [16]. Aberrant expression levels of numerous miRNAs were observed when comparing GBM tumor samples to normal brain tissue [17]. Chan et al. investigated the functional properties and expression of a single miRNA in GBM cell lines and found that inhibition of miR-21 resulted in increased apoptosis [18]. Therefore, they hypothesized that miR-21 could function as a micro-oncogene [16]. So far, about 256 miRNAs have been found to be altered in GBM, however, only a few of them, for example miR-17, miR-21, miR-93, and miR-221/222, have been intensively investigated with respect to both expression and functionality.

We observed that miR-215 is the most highly expressed miRNA in our cohort. miR-372, miR-21, mIR-10b, and miR-17 were intermediately expressed and 10 other miRNAs (miR-215, -34a, -124, -125b, -128, -137, -218, -326, -184, and -152) were lowly expressed in our cohort. These data suggest the importance of miR-215 in glioma. miR-215 was up-regulated in gastric cancer and prostate cancer using Taqman MicroRNA Assay [19–23], although it was also down-regulated in other types of cancer [23–25]. The relation of miR-215 overexpression with the progression of cancer indicates its important role as an oncogene in cancer progression [22]. Here, we observed that miR-215 was the most dramatically increased miRNA in human glioma and that the expression level of miR-215 is correlated with disease stage. These findings not only indicate the correlation of miR-215 with glioma progression, but also strongly suggest that it has an oncogenic effect.

Reports suggest that miR-215 may be a potential prognostic factor in cancer patients. miR-215 expression was greater in cervical tumors with vascular invasion and at FIGO (International Federation of Gynaecological Oncologists) stage. Additionally, the 5-year disease-free survival was lower in patients with overexpression of miR-215. Tumor size was larger in nude mice injected with HeLa-miR-215 cells than in those injected with control cells [24]. All of these findings indicate that miR-215 is a marker for the poor prognosis of cervical cancer. Downregulation of miR-215 expression also suppress gastric cancer cell invasion, and expression of miR-215 may be a potential biomarker for gastric cancer prognosis [25]. miR-215 was also linked with the development, progression, and prognosis of prostate cancer [26–28]. We observed that high miR-215 expression is correlated with poor prognosis and advanced tumor stage in glioma patients. These data suggest that miR-215 not only has an oncogenic effect, but also can be a biomarker for diagnosis and prognosis of human glioma.

Indeed, we performed multivariate and univariate analysis in the cohort and showed that miR-215 expression, recurrence or metastasis, and tumor dimension were independent poor prognostic parameters for glioma patients. These data confirmed that miR-215 is a biomarker for clinical diagnosis and prognosis of human glioma.

miR-215 decreases the expression of its targets, which include a number of cell cycle genes [29–31]. It also down-regulates the expression of Bcl-2, Zeb2, and VEGFA, to enhance apoptosis, increase the expression of E-cadherin, and decrease angiogenesis in colon cancer [32]. Overexpression of miR-215 reduced the expression of TYMS and MDM2 in renal cell carcinoma [33]. miR-215 also inhibited the expression of BMP-6 through the BMP-6/miR-192 pathway to increase cell proliferation in breast cancer [34]. miR-215 and miR-192 promoted apoptosis through the XIAP pathway in non-small cell lung cancer [35]. All these reports indicate that miR-215 exerts its biological and pathological function by regulating target gene expression. Importantly, it is reported that miR-215 is involved in the TGF-β1-induced activation of the β-catenin pathway by directly targeting and decreasing expression of CTNNBIP1 [13]. Here, we also found decreased expression of CTNNBIP1, increased β-catenin phosphorylation, and increased expression of α-SMA and fibronectin in glioma tumor tissue. We also observed the increased expression of TGF-β1 as previously reported in gliomas [12]. These data indicate that miR-215 exerts its oncogenic and metastatic effect in the tumors by directly targeting CTNNBIP1 mRNA and mediating the TGF-β1-induced activation of the β-catenin pathway in human glioma. This finding also revealed a novel mechanism underlying oncogenesis of human glioma.

In summary, we identified that miR-215 is dramatically increased in human glioma and high miR-215 expression is correlated with poor prognosis, high tumor histologic grade, and advanced tumor progression. High miR-215 expression is a potential biomarker for diagnosis and prognosis in human glioma. We also found a novel mechanism responsible for oncogenesis and metastasis of human glioma.

## MATERIALS AND METHODS

### Patients and tissue samples

This study was approved by the Institutional Review Board of Renmin Hospital, Wuhan University School of Medicine. A written informed consent was obtained from each participant in accordance with the Renmin Hospital of Wuhan University Ethics Committee. All specimens were handled and made anonymous according to the ethical and legal standards.

A total of 179 patients were analyzed in this study and underwent resection of their primary gliomas at the Department of Neurosurgery, Renmin Hospital of Wuhan University between 2005 and 2009. Resected tissue samples were immediately cut and snap-frozen in liquid nitrogen before being stored at −80°C until RNA was extracted. The patients' clinical records were reviewed to obtain data regarding gender, age, tumor dimension and grade, KPS score, and WHO classification. Fifty-six of the 179 gliomas were classified as low-grade [19 WHO I (19 pilocytic astrocytomas) and 37 WHO II (13 oligodendrogliomas, 15 oligoastrocytomas, and 9 diffuse astrocytomas)] and 123 were classified as high-grade gliomas [42 WHO III (12 oligodendrogliomas, 17 oligoastrocytomas, and 13 anaplasia astrocytomas)and 81 WHO IV (29 secondary glioblastomas and 52 primary glioblastomas)] according to WHO classification in 2007. None of the patients had received chemotherapy or radiotherapy prior to surgery. The clinicopathologic characteristics of all the patients are summarized in Table 1. Ten snap-frozen samples of non-neoplastic brain tissues from individuals undergoing decompressive craniectomy after suffering brain injuries were also included as controls. The diagnosis in all cases was confirmed by histological examination of tissue sections stained with hematoxylin and eosin (H&E).

Of the 179 glioma cases, 103 were males and 76 were females with a median age of 58.7 years ranging from 19.4 to 72.3. As part of their treatment, 87 patients received adjuvant chemotherapy and 92 received radiotherapy. At the last follow-up, 53 patients were still alive, whereas 126 had died. Overall survival time was calculated from the date of the initial surgical operation to death. Patients who died of diseases not directly related to their gliomas or due to unexpected events were excluded from this study. Several clinicopathological features, such as WHO grade, Karnofsky performance status (KPS) score, and tumor size, have been considered as important prognostic factors for gliomas [36].

### RNA isolation

Using archived FFPE tissues, 179 glioma tissues and 20 non-neoplastic brain tissues were identified using the corresponding H&E stained sections. Cores measuring 1.5 mm in diameter and 2 mm in length (approximately 5 mg) were extracted from the tumors. Subsequently, the samples were deparaffinized, hydrated, digested with proteinase K, and ultimately, total RNA was isolated using TRIzol reagent according to the manufacturer's protocol (Invitrogen, Carlsbad CA, USA).

### Real-time quantitative RT-PCR for miRNA expression

Primers for miR-215 and the internal control RNU6B gene were purchased from Ambion (Applied Biosystems, CA, USA). The cDNA synthesis was performed using the High Capacity cDNA Synthesis Kit (Applied Biosystems, CA, USA) with miRNA specific primers. Reverse transcriptase reactions contained 10 ng of total RNA, 50 nmol/l stem-loop RT primer, 1X RT buffer, 0.25 mmol/l each of deoxynucleotide triphosphates (dNTP), 3.33 U MultiScribe reverse transcriptase, and 0.25 U RNase Inhibitor. The 7.5 uL reaction volumes were incubated in Bio-Rad i-Cycler (Bio-Rad Laboratories, Hercules, CA, USA) in a 96-well plate for 30 min at 16°C, 30 min at 42°C, 5 min at 85°C, and then held at 4°C. Real-time quantitative RT-PCR (qRT-PCR) was carried out on a Roche LightCycler 480 Real-time system with miRNA specific primers by TaqMan Gene Expression Assay. The 10 X PCR mixture included 0.6 uL of RT products, 1X TaqMan Universal PCR master mix, and 1 uL of primers and probe mix of the TaqMan MicroRNA assays. Relative quantification of target miRNA expression was evaluated using the comparative cycle threshold (CT) method. The raw data were presented as the relative quantity of target miRNA, normalized with respect to RNU6B. Each sample was examined in triplicate. Mean normalized gene expression ±standard error of the mean (SEM) was calculated from independent experiments. miR-215 was considered to be highly expressed if its relative expression level was ≥5. The quantitative RT-PCR for CTNNBIP1 expression was performed similarly as above. The raw data were presented as the relative quantity of target CTNNBIP1, normalized with respect to GAPDH. The primers of CTNNBIP1 were 5′-GTGACTCTCGGAGCGGGA-3′ (forward) and 5′-GCTGGGCAGAAGTAGGAAGG-3′ (reverse). The primers of GAPDH were 5′-ATGTTGCAACCGGGAAGGAA-3′ (forward) and 5′-AGGAAAAGCATCACCCGGAG-3′ (reverse).

### Western blot

Glioma tumor tissue and adjacent normal tissue were washed with ice-cold PBS, homogenized on ice, and lysed by protein lysate (Pierce). After centrifugation, the protein concentration was measured by BCA protein assay kit (Pierce). Fifty micrograms of lysate was used for western blot. Briefly, whole cell protein extracts were separated on 10% SDS-PAGE and electroblotted onto a nitrocellulose membrane (Amersham). The blocked membrane was incubated first with anti-DKC1 polyclonal mouse antibody (Santa Cruz) or anti-beta-actin antibody (Sigma) and then with rabbit anti-mouse antibody conjugated with horseradish peroxidase (Sigma). The signal was detected using BM Blue POD Substrate (Roche). The following antibodies were used: anti-CTNNBIP1 antibody (1:200; Santa Cruz), anti-β-Catenin (t-β-Catenin) antibody (1:200; Product code: ab47426; Abcam), anti-β-Catenin (phospho T41 + S45, p-β-Catenin) antibody (1:200; Product code: ab81305; Abcam), anti-α-SMA antibody (1:200; Sigma, St. Louis, MO), and anti-fibronectin antibody (1:200; Santa Cruz). Anti-β-actin antibody (1:1000; Sigma) was used as a loading control.

### Statistical analysis

All computations were carried out using GraphPad Prism version 6.0 for Windows (GraphPad Software, Inc., USA). Data were expressed as mean ± SEM. The analysis of variance (ANOVA) was used to determine statistical differences among the groups. The Kaplan-Meier method was used to estimate survival rates, and the log-rank and Wilcoxon rank sum tests were used to assess survival differences between groups. The Cox proportional hazards model for multivariate survival analysis was used to assess predictors related to survival. Comparison of quantitative variables was done by Pearson correlation method. Differences were considered statistically significant when *P* was < 0.05.
